# Factors Associated With the Use of Telehealth in Middle-Aged and Older Adults During the COVID-19 Pandemic

**DOI:** 10.1093/geroni/igab046.3297

**Published:** 2021-12-17

**Authors:** Yan Du, Rumei Yang, Qingwen Xu, Bo Xie

**Affiliations:** 1 School of Nursing, University of Texas Health Science Center at San Antonio, San Antonio, Texas, United States; 2 Nanjing Medical University, Nanjing, Jiangsu, China (People's Republic); 3 New York University, New York University, New York, United States; 4 The University of Texas at Austin, Austin, Texas, United States

## Abstract

The COVID-19 pandemic has promoted the adoption and use of telehealth, particularly in the early months of the pandemic. However, people with diverse characteristics may, or may not, be able to use telehealth, presenting digital divide in health care and potential health equity-related issues. This study aimed to assess the use of telehealth among middle-aged and older adults during COVID-19, and to explore factors associated with their telehealth utilization. We used publicly available data from the California Health Interview Survey collected during January 2019 and December 2020 (N=15, 279; mean age= 64.23±11.59; female: 52.7%). Approximately 11.0% of the sample used telehealth at least once. Bivariate and multivariate logistical regression analyses found that, compared with non-users, telehealth users were more likely to be having higher numbers of chronic conditions, with self-reported mental distress, living in urban areas, born in the US, with higher English proficiency, higher education, and having higher incomes. Age, race/ethnicity, and gender were not significantly correlated with telehealth usage. Logistic regression revealed that having mental distress (OR=1.48, 95% CI=1.29-1.71, p<0.01), more chronic conditions (OR=1.48, 95% CI=1.29-1.71, p<0.001) and living in an urban area (OR=1.93, 95% CI=1.36-2.74, p<0.001) were independently related to telehealth use. These findings suggest that telehealth, while being beneficial during the pandemic, might also introduce new challenges that exacerbate existing health inequity and disparities. Policy and community-based interventions are needed to promote the use of telehealth among middle-aged and older adults with diverse characteristics.

